# Inflammatory Indices in Preterm Infants with Hemodynamically Significant Patent Ductus Arteriosus: Early Postnatal Patterns and Treatment Response

**DOI:** 10.3390/children13070945

**Published:** 2026-07-18

**Authors:** Demet Türkeli, Ilkay Er, Hikmet Kıztanır, Medeni Arpa

**Affiliations:** 1 Department of Pediatrics, Faculty of Medicine, Recep Tayyip Erdogan University, 53200 Rize, Turkey; demet.dr101@gmail.com; 2 Department of Pediatrics, Division of Neonatology, Faculty of Medicine, Recep Tayyip Erdogan University, 53200 Rize, Turkey; 3 Department of Pediatrics, Division of Pediatric Cardiology, Faculty of Medicine, Recep Tayyip Erdogan University, 53200 Rize, Turkey; hikmet.kiztanir@erdogan.edu.tr; 4 Department of Medical Biochemistry, Faculty of Medicine, Recep Tayyip Erdogan University, 53200 Rize, Turkey; medeni.arpa@erdogan.edu.tr

**Keywords:** patent ductus arteriosus, hemodynamically significant patent ductus arteriosus, preterm infants, inflammation, complete blood count-derived indices, pan-immune-inflammation value, temporal patterns, treatment response

## Abstract

**Background**: Inflammatory mechanisms may contribute to persistent ductal patency in preterm infants with hemodynamically significant patent ductus arteriosus (hsPDA). This study evaluated early postnatal temporal patterns of complete blood count (CBC)-derived inflammatory indices in relation to ductal status and pharmacologic treatment response. **Methods**: This retrospective single-center study included preterm infants born at <34 weeks’ gestation who received pharmacologic treatment for hsPDA. Hematologic parameters and CBC-derived inflammatory indices, including neutrophil-to-lymphocyte ratio (NLR), monocyte-to-lymphocyte ratio (MLR), platelet-to-lymphocyte ratio (PLR), systemic immune-inflammation index (SII), systemic inflammation response index (SIRI), pan-immune inflammation value (PIV), and immature granulocyte (IG) parameters, were assessed on postnatal days 1, 3, and 7. **Results**: The final cohort included 71 preterm infants with hsPDA. Over the first postnatal week, NLR and PLR peaked on day 3, MLR remained elevated, and PIV increased toward day 7. SII and SIRI showed significant temporal variation, whereas IG parameters did not. Among longitudinal changes, only ΔPIV, calculated as day 3 minus day 7 values, differed by treatment response, with a greater increase in PIV observed among non-responders. ΔPIV yielded limited discrimination for non-response (AUC = 0.646; sensitivity, 92%; specificity, 37%; cut-off, ≤110.4). Birth weight and gestational age were positively correlated with several hematologic parameters and inflammatory indices, whereas ductal status showed inverse associations with IG% on day 1 and with monocyte count and MLR on day 3. After adjustment for maturational factors and treatment type, the ΔPIV–response association was attenuated, without improved discrimination. **Conclusions**: CBC-derived inflammatory indices demonstrated distinct early postnatal patterns in infants with hsPDA. ΔPIV may serve as a hypothesis-generating longitudinal marker of treatment response rather than a stand-alone predictor and warrants further validation.

## 1. Introduction

The ductus arteriosus (DA) is a vital fetal shunt that normally closes after birth [1]. Failure of closure, particularly in preterm infants, results in patent ductus arteriosus (PDA), which is associated with increased pulmonary blood flow, reduced systemic perfusion, and major complications of prematurity [2,3]. Although ductal closure is classically attributed to oxygen-mediated smooth muscle constriction, reduced prostaglandin signaling, and subsequent vascular remodeling of the ductal wall [1,3], accumulating evidence suggests that inflammatory mechanisms may also modulate ductal patency in preterm infants [4,5,6,7].

Systemic inflammation is accompanied by coordinated changes in circulating immune cells, including neutrophilia, lymphopenia, monocyte mobilization, and platelet activation, which may contribute to vascular inflammation and endothelial dysfunction [8,9]. Accordingly, complete blood count (CBC)-derived inflammatory indices, such as the neutrophil-to-lymphocyte ratio (NLR), monocyte-to-lymphocyte ratio (MLR), and platelet-to-lymphocyte ratio (PLR), have been widely investigated as markers of inflammatory activity [9,10,11]. More recently, composite indices, including the systemic immune-inflammation index (SII), systemic inflammation response index (SIRI), and pan-immune-inflammation value (PIV), have been introduced to capture the integrated inflammatory burden and its association with clinical outcomes across diverse settings [9,10,11,12]. In parallel, immature granulocyte (IG) parameters may provide additional information on the systemic inflammatory response by reflecting bone marrow activation and the release of immature myeloid cells into the circulation [13,14].

In neonatal research, CBC-derived inflammatory indices have been increasingly explored in conditions such as early-onset sepsis, transient tachypnea of the newborn, bronchopulmonary dysplasia, and retinopathy of prematurity [15,16,17,18,19,20,21,22]. CBC-derived inflammatory markers have also been investigated in congenital cardiovascular diseases, primarily in congenital heart disease and perioperative cardiac surgery settings. Among these, NLR has been the most frequently studied marker and has been associated with cyanotic status, pulmonary hypertension, and postoperative outcomes after congenital heart surgery [23,24]. PLR has also been evaluated, especially in univentricular patients undergoing Fontan palliation [25], whereas more integrated indices such as SII have only recently been explored in children undergoing surgery for congenital heart disease [26].

Emerging evidence suggests that certain inflammatory indices may be associated with the presence or clinical course of hemodynamically significant PDA (hsPDA) [4,5,7]. To our knowledge, their longitudinal patterns in hsPDA, particularly in relation to treatment response, remain insufficiently characterized. This study aimed to evaluate CBC-derived inflammatory indices, as well as hematologic parameters, in preterm infants treated for hsPDA, focusing on their early postnatal temporal patterns in relation to ductal status and treatment response.

## 2. Materials and Methods

### 2.1. Study Design and Population

This single-center retrospective study was conducted in the Neonatal Intensive Care Unit (NICU) at Recep Tayyip Erdogan University Faculty of Medicine between January 2023 and December 2025. Medical records of preterm infants admitted during this period were reviewed. Eligible infants were those born at <34 weeks’ gestation who underwent echocardiographic evaluation at 48–72 h of life and met the diagnostic criteria for hsPDA. Given the current trend toward individualized pharmacologic treatment of PDA, this gestational age cutoff was selected to focus on infants at higher risk for clinically significant ductal patency and to reduce maturational heterogeneity [2,27,28].

To minimize potential confounders affecting ductal physiology or inflammatory parameters, exclusion criteria included exposure to antenatal corticosteroids; maternal clinical chorioamnionitis; neonatal sepsis or elevated C-reactive protein levels (CRP > 10 mg/L); major congenital anomalies or chromosomal abnormalities; congenital heart disease, including ductus-dependent lesions; pulmonary hypertension; perinatal asphyxia; primary or secondary thrombocytopenia (<100 × 10^3^/µL); and incomplete clinical or echocardiographic data [1,2,27].

Clinical chorioamnionitis was defined according to the criteria described by Gibbs et al. as maternal fever accompanied by at least two of the following findings: maternal tachycardia, fetal tachycardia, uterine tenderness, foul-smelling amniotic fluid, or ma-ternal leukocytosis [29].

The study was conducted in accordance with the Declaration of Helsinki and approved by the institutional ethics committee. The requirement for informed consent was waived because of its retrospective design.

### 2.2. Echocardiographic Evaluation and Management of PDA

Echocardiographic evaluations were performed after the first 48 h of life by an experienced pediatric cardiologist using a Vivid S6 echocardiography system equipped with a 10S transducer (GE Healthcare, Milwaukee, WI, USA). hsPDA was defined by the presence of at least two clinical signs and at least one echocardiographic criterion. Clinical signs included hyperdynamic precordium, continuous murmur, tachycardia, hypotension, oliguria, widened pulse pressure, and increased need for respiratory support and/or supplemental oxygen. Echocardiographic criteria included a ductal diameter ≥1.5 mm, a left atrium-to-aortic root ratio ≥1.5, and/or absent or reversed diastolic flow in the abdominal aorta [2,27,28]. All measurements were performed according to standard neonatal echocardiographic guidelines.

Pharmacologic treatment for ductal closure consisted of ibuprofen or paracetamol administered according to clinical indications. Ibuprofen was considered the first-line pharmacologic option, whereas paracetamol was selected in the presence of contraindications to ibuprofen, including active bleeding, thrombocytopenia, coagulopathy, necrotizing enterocolitis, or renal impairment [2,27,28,30]. Ibuprofen was administered as a loading dose of 10 mg/kg, followed by two successive doses of 5 mg/kg at 24 h intervals. Paracetamol was administered at a dose of 15 mg/kg every 6 h, typically for 3 days, with treatment extended up to 7 days when clinically indicated [27,28].

Follow-up echocardiography was performed approximately 72 h after initiation of pharmacologic therapy to reassess ductal status. Resolution of hsPDA at this first evaluation was considered treatment response, whereas persistence of hsPDA defined non-response. In non-responders, subsequent pharmacologic management was individualized according to clinical status. Treatment options included standard- or high-dose ibuprofen and, in selected cases, combined ibuprofen–paracetamol therapy. Surgical ligation was considered after two or three unsuccessful pharmacologic courses [27,28].

### 2.3. Blood Sampling and Analysis of CBC-Derived Inflammatory Indices

As part of routine clinical care, CBC analyses were performed on peripheral blood samples collected into EDTA-anticoagulated tubes and processed using an automated hematology analyzer (Mindray BC-6000; Mindray Bio-Medical Electronics Co., Ltd., Shenzhen, China). Leukocyte, neutrophil, monocyte, lymphocyte, platelet, and IG counts (×10^3^/µL), as well as IG percentage (IG%), were recorded. CBC-derived systemic inflammatory indices were calculated as follows: NLR = N/L, PLR = P/L, MLR = M/L, SII = P × N/L, SIRI = N × M/L, and PIV = P × N × M/L [10,11].

CBC parameters measured on postnatal days 1, 3, and 7 were included in the analysis. Day 1 values, obtained within the first 6 h of life, served as early postnatal baseline measurements. Day 3 values, obtained at 48–72 h of life before the initiation of pharmacologic therapy for hsPDA, were considered pre-treatment measurements. Day 7 values were assessed as post-treatment follow-up measurements. Variables were labeled according to the corresponding sampling day, for example, Leukocyte-1, Leukocyte-3, Leukocyte-7, NLR-1, NLR-3, and NLR-7. Changes between day 3 and day 7 values (Δ) were calculated as day 3 minus day 7 measurements.

### 2.4. Statistical Analyses

All statistical analyses were conducted using IBM SPSS Statistics version 29.0 (IBM Corp., Armonk, NY, USA) and MedCalc Statistical Software version 19.1.3 (MedCalc Software bv, Ostend, Belgium; https://www.medcalc.org, accessed on 10 February 2026). Normality of continuous variables was assessed using the Kolmogorov–Smirnov test. Descriptive statistics are presented as mean ± standard deviation (SD) for normally distributed continuous variables, median (minimum–maximum) for non-normally distributed variables, and frequency with percentage for categorical variables.

Comparisons between independent groups were performed using the independent-samples *t*-test or Mann–Whitney U test, as appropriate. Paired comparisons between two related measurements were performed using the paired-samples *t*-test or Wilcoxon signed-rank test. Categorical variables were compared using the chi-square test, with Fisher’s exact test applied when expected cell frequencies were less than five. Temporal changes in hematologic parameters and inflammatory indices across postnatal days 1, 3, and 7 were evaluated using repeated-measures analysis of variance (ANOVA) for normally distributed data, followed by Bonferroni-adjusted post hoc pairwise comparisons. For non-normally distributed repeated measures, the Friedman test was used, followed by Bonferroni-adjusted Wilcoxon signed-rank tests for pairwise comparisons. Associations between continuous variables were assessed using Spearman’s rank correlation coefficient.

Receiver operating characteristic (ROC) curve analysis was performed to evaluate the discriminative performance of selected parameters. The area under the curve (AUC) was calculated, and optimal cut-off values were determined using the Youden index. Multivariable logistic regression models were constructed where appropriate, with birth weight, gestational age, and treatment type (paracetamol vs. ibuprofen) included as clinically relevant covariates. Adjusted odds ratios (ORs) with 95% confidence intervals (CIs) were reported when applicable. To assess whether a selected parameter provided incremental discriminative value, a “basic” logistic regression model including birth weight, gestational age, and treatment type was first developed. A corresponding “full” model was created by additionally incorporating the selected parameter into the basic model. Predicted probabilities from each model were used to generate ROC curves, and the discriminative performance of the two models was compared using DeLong’s test for correlated ROC curves. All statistical tests were two-tailed, and a p-value < 0.05 was considered statistically significant.

## 3. Results

### 3.1. Study Design and Patient Selection

**Figure 1:**
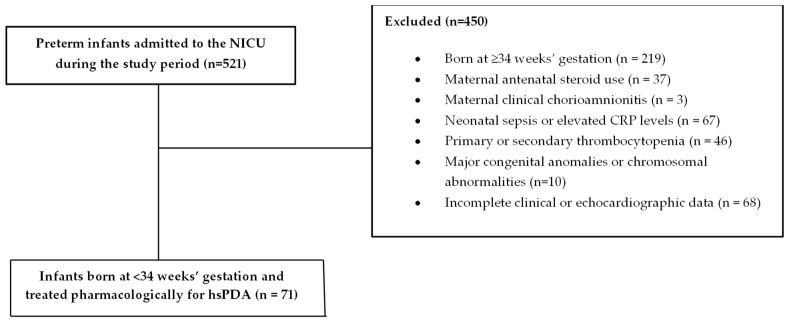
Flow diagram of patient selection for the final study cohort.

During the study period, 521 preterm infants admitted to the NICU were screened. After application of the predefined inclusion and exclusion criteria, 71 infants born at <34 weeks’ gestation with hsPDA who received pharmacologic treatment comprised the final study cohort. The cohort selection process is illustrated in Figure 1.

Baseline characteristics of the final cohort were as follows: the median birth weight was 1400 g (range, 410–2200), and the median gestational age was 30 weeks (range, 22–33.4). Male infants accounted for 36 cases (50.7%).

### 3.2. Temporal Changes in Hematologic Parameters During the First Postnatal Week

Temporal changes in hematologic parameters on postnatal days 1, 3, and 7 in preterm infants treated for hsPDA are presented in Table 1.

Total leukocyte counts varied significantly over time (*p* = 0.010), mainly reflecting higher day 7 values compared with day 3. Monocyte counts increased significantly over time (*p* < 0.001), driven by higher day 7 values compared with days 1 and 3. Lymphocyte counts decreased from day 1 to day 3 and recovered by day 7 (*p* < 0.001), with significant day 1–day 3 and day 3–day 7 differences. In contrast, neutrophil and platelet counts showed no significant temporal variation.

### 3.3. Temporal Changes in Inflammatory Indices and Immature Granulocyte Parameters During the First Postnatal Week

In parallel with hematologic changes, several inflammatory indices showed significant temporal variation during the first postnatal week (Table 2). NLR and PLR increased from day 1 to day 3 and declined by day 7 (both *p* < 0.001), with significant day 1–day 3 and day 3–day 7 differences. MLR also increased by day 3 and remained elevated on day 7 compared with baseline (*p* < 0.001). PIV increased progressively toward day 7 (*p* < 0.001), although the day 3–day 7 difference did not remain significant after adjustment. SII and SIRI showed significant temporal variation (*p* = 0.002 and *p* < 0.001, respectively), with early increases followed by relative stabilization. In contrast, absolute IG count and IG% did not vary significantly over time.

### 3.4. Temporal Changes in Hematologic Parameters and Inflammatory Indices by Treatment Response

Paracetamol was the most commonly used initial pharmacologic agent, accounting for 94.4% of cases. Persistent hsPDA at the first follow-up echocardiographic evaluation was observed in 25 infants (35.2%), who were classified as non-responders; the remaining 46 infants (64.8%) were responders. Birth weight and gestational age were numerically lower in non-responders than in responders, although the differences did not reach statistical significance (*p* = 0.093 and *p* = 0.068, respectively). The median birth weight was 1020 g (410–2000) in non-responders and 1412 g (555–2200) in responders, while the corresponding median gestational ages were 29.4 weeks (23.2–31.2) and 30.8 weeks (24.6–33.4), respectively. Three non-responders ultimately underwent surgical ligation after hsPDA persisted despite the initial pharmacologic course and two additional treatment courses.

Comparative analyses of hematologic parameters and inflammatory indices according to treatment response are presented in Table 3. No significant between-group differences were observed on postnatal days 3 or 7 (all *p* > 0.05). However, distinct within-group temporal patterns were observed pre-treatment day 3 to post-treatment day 7. In the non-responder group, leukocyte, monocyte, and lymphocyte counts increased significantly over time (*p* = 0.005, *p* = 0.006, and *p* = 0.015, respectively). These changes were accompanied by significant increases in PIV (*p* < 0.001), SIRI (*p* = 0.010), absolute IG count (*p* = 0.032), and IG% (*p* = 0.044), whereas neutrophil count, platelet count, NLR, MLR, PLR, and SII remained unchanged. In the responder group, a more selective temporal pattern was observed: monocyte and lymphocyte counts increased between day 3 and day 7 (*p* = 0.010 and *p* < 0.001, respectively), while NLR and PLR decreased significantly (both *p* < 0.001). Other hematologic parameters and inflammatory indices remained stable.

### 3.5. Comparison of Δ (Day 3–Day 7) Hematologic Parameters and Inflammatory Indices by Treatment Response

Among all Δ parameters calculated as day 3 minus day 7 measurements, only ΔPIV differed significantly between responders and non-responders (*p* = 0.044). Changes in the remaining hematologic parameters and inflammatory indices did not differ significantly between groups, although ΔSIRI approached but did not reach statistical significance (*p* = 0.076) (Table 4).

**Figure 2:**
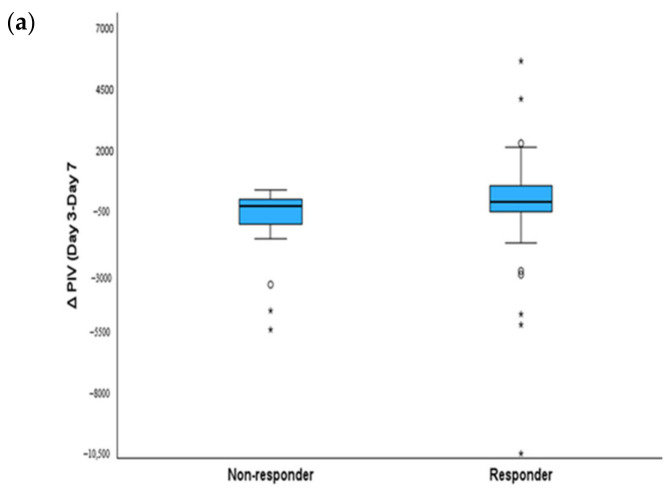

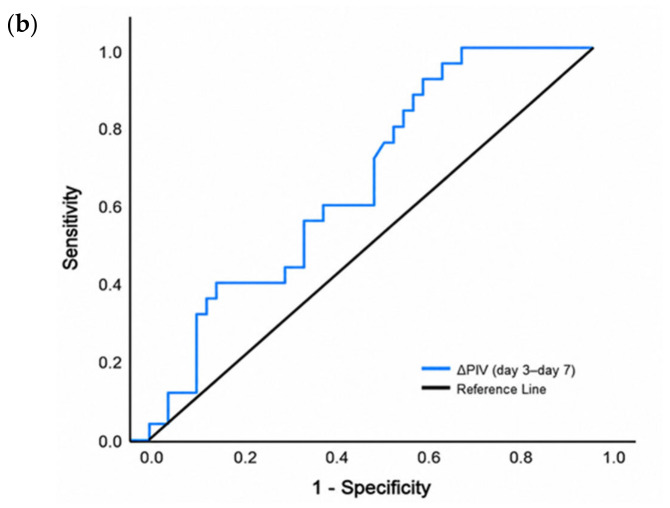
(**a**) Distribution of ΔPIV (day 3–day 7) values according to treatment response. ^0^: mild outliers, defined as values lying between 1.5× and 3× the interquartile range (IQR) from the quartiles; *: extreme outliers, defined as values beyond 3× IQR. (**b**) ROC curve of ΔPIV (day 3–day 7) for identifying treatment non-response in preterm infants with hsPDA. ΔPIV was calculated as the postnatal day 3 value minus the postnatal day 7 value. PIV: pan-immune-inflammation value; hsPDA: hemodynamically significant patent ductus arteriosus.

Because Δ values were defined as day 3 minus day 7 measurements, negative ΔPIV values indicate an increase in PIV from day 3 to day 7. Accordingly, the lower, more negative ΔPIV values observed in non-responders suggest a greater increase in PIV over time, whereas ΔPIV values in responders were centered closer to zero. This between-group difference in ΔPIV is illustrated in Figure 2a. ROC analysis demonstrated modest discriminative performance of ΔPIV for identifying treatment non-response, with an AUC of 0.646. At a cut-off value of ≤110.4, ΔPIV showed a specificity of 37% and a sensitivity of 92% (Figure 2b, Table 5).

### 3.6. Correlations Between Hematologic Indices and Ductal Status

Correlation analyses of hematologic parameters and inflammatory indices with hsPDA status, birth weight, and gestational age on pre-treatment postnatal day 3 are presented in Table 6. hsPDA status showed weak but statistically significant inverse correlations with monocyte count (r = −0.258, *p* = 0.030) and MLR (r = −0.259, *p* = 0.029), whereas no significant associations were observed with the remaining hematologic parameters or inflammatory indices. In addition, hsPDA status was not significantly correlated with birth weight or gestational age.

By comparison, birth weight showed significant positive correlations with leukocyte, neutrophil, lymphocyte, and platelet counts, as well as with PIV, SII, SIRI, and IG count. A similar pattern was observed for gestational age, which was positively correlated with leukocyte, neutrophil, lymphocyte, and platelet counts, in addition to PIV, SII, and SIRI.

A separate correlation analysis using early postnatal measurements on postnatal day 1 revealed a weak inverse correlation between hsPDA status and IG% (r = −0.251, *p* = 0.035), whereas no significant correlations were observed with the remaining parameters.

Overall, hsPDA-related correlations were modest in magnitude and differed according to postnatal timing.

### 3.7. Multivariable and Incremental Analyses of Δ Parameters in Relation to Treatment Response

The evaluated Δ (Day 3–Day 7) parameters were further examined using logistic regression models adjusted for birth weight, gestational age, and treatment type. After adjustment, the associations with treatment non-response were attenuated and did not reach statistical significance, including for ΔPIV (adjusted OR = 0.968, 95% CI: 0.920–1.018, *p* = 0.206) (Table 7).

To assess the incremental discriminative value of ΔPIV, a basic model including birth weight, gestational age, and treatment type was compared with a full model additionally incorporating ΔPIV. The full model showed a numerically higher AUC than the basic model (0.652 [95% CI: 0.530–0.761] vs. 0.618 [95% CI: 0.495–0.731]); however, this difference was not statistically significant (ΔAUC = 0.034 [95% CI: −0.035 to 0.103], z = 0.962, *p* = 0.336).

## 4. Discussion

In this cohort of preterm infants with hsPDA, CBC-derived inflammatory indices followed distinct temporal trajectories during the first postnatal week. NLR and PLR peaked on day 3, whereas MLR remained elevated and PIV increased toward day 7; SII and SIRI also showed significant temporal variation. These patterns were accompanied by hematologic changes consistent with a transition from early lymphocyte suppression toward sustained monocyte-related inflammatory activity. Among the longitudinal changes evaluated according to treatment response, ΔPIV, calculated as day 3 minus day 7 values, was the only parameter associated with pharmacologic non-response in unadjusted comparisons. More negative ΔPIV values observed in non-responders indicate a greater increase in PIV from day 3 to day 7, suggesting that longitudinal inflammatory changes may accompany persistent ductal patency. However, the overall discriminative performance of ΔPIV was limited (AUC = 0.646), with low specificity despite high sensitivity. Correlation analyses further demonstrated selective associations with ductal status, including inverse relationships for IG% on day 1 and for monocyte count and MLR on day 3. In addition, birth weight and gestational age were positively correlated with multiple hematologic parameters and inflammatory indices, underscoring the influence of gestational maturity when interpreting CBC-derived inflammatory indices in this population. After adjustment for these maturational variables and treatment type, ΔPIV was not independently associated with treatment non-response (adjusted OR = 0.968, 95% CI: 0.920–1.018, *p* = 0.206). Adding ΔPIV to the basic model also did not significantly improve discriminative performance (ΔAUC = 0.034, *p* = 0.336). These findings support a cautious interpretation of ΔPIV as an exploratory longitudinal marker rather than an independent biomarker of treatment response.

Our findings support the view that systemic inflammatory activity may contribute to ductal persistence and treatment non-response in preterm infants with hsPDA, beyond the classical mechanisms of postnatal ductal closure. Perinatal inflammatory exposure may induce a sustained inflammatory response, particularly in preterm infants with immature immune regulation [31,32]. Experimental and clinical evidence further suggests that proinflammatory cytokine activation may interfere with ductal regulatory pathways and promote persistent patency [6,33]. Although chorioamnionitis has been widely investigated as a marker of antenatal inflammation, its association with PDA remains inconsistent across studies [34,35,36]. This inconsistency may reflect differences in gestational maturity, inflammatory phenotype, and the limited concordance between placental inflammation and fetal inflammatory response [6,34]. In our cohort, infants exposed to maternal clinical chorioamnionitis, as well as those with neonatal sepsis or elevated CRP levels, were excluded, reducing the likelihood that the observed hematologic patterns were primarily attributable to overt infectious inflammation.

At the cellular level, systemic inflammation is characterized by coordinated changes across neutrophil, lymphocyte, monocyte, and platelet compartments. Among these changes, neutrophilia and lymphocytopenia may result from enhanced neutrophil mobilization together with lymphocyte redistribution and apoptosis mediated by neuroendocrine and cytokine-related pathways. In parallel, platelet activation and cytokine-mediated thrombopoietic responses may amplify inflammatory processes through effects on microcirculation, vascular permeability, and aggregation. Monocytosis may similarly reflect sustained inflammatory signaling through activation of the monocyte–macrophage axis [6,10,11]. Together, these cellular changes provide the biological basis for CBC-derived inflammatory indices, including NLR, MLR, PLR, SII, SIRI, and PIV, which reflect the interplay of multiple immune cell lineages. Although much of the evidence regarding these indices is derived from adult studies, emerging neonatal data suggest that they may also have clinical relevance across infectious, inflammatory, respiratory, and metabolic conditions and related outcomes [15,16,17,18,19,20,21,22]. Given their availability, reproducibility, and cost-effectiveness, these indices may help characterize the evolving inflammatory milieu associated with hsPDA [7].

Despite their biological plausibility and practical advantages, the clinical relevance of CBC-derived inflammatory indices among preterm infants with hsPDA remains incompletely defined. Matsushita et al. identified an “inflamed” hsPDA subphenotype characterized by higher leukocyte, neutrophil, and NLR values, alongside a second subgroup primarily associated with respiratory acidosis [4]. Conversely, Temel et al. reported no significant difference in NLR between infants with and without PDA, although NLR showed a positive correlation with birth weight [37]. In our cohort, birth weight and gestational age were associated with several hematologic parameters and composite inflammatory indices, whereas NLR showed neither a maturational association nor a selective relationship with hsPDA status, despite increasing during the early postnatal period and peaking on day 3. These findings suggest that NLR may have limited utility as an isolated marker of PDA status but could still help characterize early inflammatory profiles across heterogeneous hsPDA presentations.

Karabulut et al. reported higher PLR values on postnatal days 1, 2, 3, and 7 in infants with hsPDA, with the strongest discriminative performance observed on day 3 (AUC = 0.923), accompanied by lower lymphocyte counts [5]. Our longitudinal assessment also demonstrated a day-3 increase in PLR, which may support the relevance of early postnatal lymphocyte-related inflammatory changes in hsPDA. In addition, MLR remained persistently elevated, while hsPDA status was inversely correlated with IG% on day 1 and with monocyte count and MLR on day 3. Given the nonspecific nature of IG parameters and their sensitivity to perinatal inflammatory exposure, hematopoietic stress, and early postnatal adaptation, the day-1 IG% finding should be interpreted cautiously [13,38]. The day-3 associations with monocyte count and MLR may indicate a time-dependent contribution of monocyte-related inflammatory activity, although the weak magnitude of these correlations limits the ability to draw causal inferences.

As a composite index integrating neutrophil, monocyte, lymphocyte, and platelet counts, PIV may reflect systemic inflammatory burden [11]. A recent study by Çakır et al. evaluated systemic inflammatory indices during the first 24 h of life and reported that PIV, but not NLR, MLR, PLR, SII, or SIRI, was significantly associated with hsPDA, with an AUC of 0.618 [7]. Together with these observations, the present results suggest that serial PIV assessment, particularly ΔPIV, may provide additional insight into evolving inflammatory activity related to persistent ductal patency and pharmacologic treatment response. However, the discriminatory capacity of ΔPIV was limited, as reflected by an AUC of 0.646, restricting its potential clinical utility. Moreover, the observed association was not independent of maturational and treatment-related factors, indicating that ΔPIV should be interpreted within the broader clinical context.

### Strengths and Limitations

This study has several strengths. First, it provides a longitudinal assessment of multiple CBC-derived inflammatory indices during the first postnatal week, allowing a detailed characterization of evolving inflammatory patterns among preterm infants with hsPDA. To the best of our knowledge, this is one of the few studies to examine these temporal changes in relation to treatment response. In addition, the inclusion of composite indices such as SII, SIRI, and PIV enabled a more integrative evaluation of inflammatory burden beyond conventional hematologic parameters. The longitudinal assessment of these indices, particularly ΔPIV, provided additional insight into inflammatory profiles associated with pharmacologic treatment response. Together with the findings of Çakır et al. [7], the present results further suggest that PIV deserves consideration as a potentially informative CBC-derived inflammatory index in infants with hsPDA. The addition of multivariable and incremental analyses enabled these observations to be interpreted within the context of maturational factors and treatment-related heterogeneity.

However, several limitations should be acknowledged. The retrospective, single-center design may reduce generalizability, a concern also noted in previous studies evaluating CBC-derived inflammatory indices in hsPDA [4,7,37]. The relatively small sample size, particularly the small number of treatment non-responders (*n* = 25), may reduce statistical power and increase the risk of model instability. The stringent exclusion criteria were applied to minimize confounding from conditions that could independently affect inflammatory indices, but may also constrain external validity. In addition, an appropriate comparison group without hsPDA could not be included in the retrospective setting, as serial CBC measurements at matched time points throughout the first postnatal week were not routinely available for clinically stable preterm infants. The specificity of these temporal patterns for hsPDA requires prospective confirmation. A further consideration is the potential for type I error arising from the evaluation of multiple biomarkers across several time points and analytical comparisons. Although Bonferroni correction was applied to repeated within-parameter comparisons across postnatal days, it may not fully account for multiplicity across biomarkers and secondary analyses. Thus, findings with borderline statistical significance should be regarded as preliminary pending external replication.

Although infants with neonatal sepsis or elevated CRP levels were excluded to minimize the influence of overt infectious inflammation, the observed increase in WBC, monocyte count, absolute IG count, and IG% among non-responders raises the possibility of an unrecognized or evolving infection that cannot be fully excluded in a retrospective design. Alternatively, this myeloid-oriented pattern may partly reflect greater developmental immaturity, given the influence of gestational and postnatal age on hematologic and innate immune profiles, while persistent hsPDA-related hemodynamic stress may further contribute to sustained inflammatory activity [6,39,40].The absence of cytokine measurements, such as IL-6, IL-8, and TNF-α, also precluded direct confirmation of the underlying inflammatory pathways [6].

Another important limitation is that day 7 CBC measurements were obtained after the initiation of pharmacologic treatment for hsPDA. The greater increase in PIV observed among non-responders cannot be attributed to a single mechanism and may reflect persistent ductal patency, underlying clinical status, or their combined effects. Pharmacologic agents may have influenced the hematologic components used to calculate PIV; NSAIDs can affect platelet function through cyclooxygenase inhibition and modulate neutrophil-related inflammatory responses, whereas paracetamol has been reported to exert variable effects on platelet function [41,42,43]. The mechanisms underlying the hematologic changes observed between days 3 and 7, as well as their clinical significance, remain uncertain in this retrospective study.

In addition to these potential treatment-related effects, treatment selection bias also warrants consideration. Although ibuprofen was regarded as the first-line pharmacologic option in our unit, paracetamol was used initially in most infants because of contraindications to ibuprofen or clinical concerns regarding NSAID-related adverse effects [30,44]. As therapy was not assigned according to a standardized protocol, residual confounding by indication related to illness severity, unmeasured comorbidities, and other determinants of therapeutic choice cannot be fully excluded, despite adjustment for treatment type, birth weight, and gestational age. The predominance of paracetamol may therefore reduce the applicability of these findings to populations with different treatment practices.

## 5. Conclusions

CBC-derived inflammatory indices demonstrated distinct temporal patterns during the early postnatal period in preterm infants with hsPDA, including day-3 increases in NLR and PLR, sustained elevation of MLR, and an increase in PIV toward day 7. Among the longitudinal changes evaluated, ΔPIV (day 3–day 7) was the only parameter associated with treatment response in unadjusted analyses, with greater increases in PIV observed among non-responders, suggesting that longitudinal inflammatory changes may accompany persistent ductal patency. However, ΔPIV demonstrated only modest discriminative ability and did not provide significant incremental value beyond birth weight, gestational age, and treatment type. Although ΔPIV may reflect evolving inflammatory dynamics related to treatment response, it should be considered a hypothesis-generating marker rather than an independent predictor of pharmacologic non-response.

Collectively, the present results suggest that a longitudinal multi-index approach may provide a more integrated understanding of inflammatory processes in hsPDA while accounting for early postnatal maturation and treatment-related heterogeneity. Further prospective, multicenter studies are warranted to validate these observations and clarify the clinical utility of longitudinal CBC-derived inflammatory indices in hsPDA.

## Figures and Tables

**Table 1:** Temporal Changes in Hematologic Parameters on Postnatal Days 1, 3, and 7 in Preterm Infants Treated for hsPDA.

Parameters	PDA-Treated Infants, *n* = 71 ^1^	*p* Value *
Leukocyte-1 (10^3^/µL)	10.520 (2.400–33.520)	**0.01** ^#^
Leukocyte-3 (10^3^/µL)	8.760 (2.270–68.930)	
Leukocyte-7 (10^3^/µL)	11.360 (4.620–48.020)	
Neutrophil-1 (10^3^/µL)	3.970 (470–28.690)	0.355
Neutrophil-3 (10^3^/µL)	4.910 (670–32.670)	
Neutrophil-7 (10^3^/µL)	4.520 (1490–35.380)	
Monocyte-1 (10^3^µ/L)	760 (20–4.100)	**<0.001** ^¥,^**^#^**
Monocyte-3 (10^3^µ/L)	880 (30–76.00)	
Monocyte-7 (10^3^µ/L)	1.480 (40–6.210)	
Lymphocyte-1 (10^3^µ/L)	4.919 ± 2.828	**<0.001** ^&,^**^#^**
Lymphocyte-3 (10^3^µ/L)	2.940 ± 1.309	
Lymphocyte-7 (10^3^µ/L)	4.147 ± 1.325	
Platelet-1 (10^3^/µL)	244.394 ± 73.084	0.56
Platelet-3 (10^3^/µL)	231.214 ± 88.334	
Platelet-7 (10^3^/µL)	243.408 ± 84.788	

^1^ Data are presented as mean ± standard deviation (SD) or median (minimum–maximum), as appropriate. hsPDA: hemodynamically significant patent ductus arteriosus. Suffixes “-1,” “-3,” and “-7” denote measurements obtained on postnatal days 1, 3, and 7, respectively. Repeated-measures ANOVA with Bonferroni-adjusted post hoc comparisons were used to evaluate temporal changes across postnatal days 1, 3, and 7. ^&^ indicates comparison between day 1 and day 3; ^#^ indicates comparison between day 3 and day 7; ^¥^ indicates comparison between day 1 and day 7. * Bold *p*-values indicate statistical significance (*p* < 0.05).

**Table 2:** Temporal Changes in Inflammatory Indices and Immature Granulocyte Parameters on Postnatal Days 1, 3, and 7 in Preterm Infants Treated for hsPDA.

Parameters	PDA-Treated Infants, *n* = 71 ^1^	* *p* Value
NLR-1	1.24 (0.10–58.84)	**<0.001** ^&,^**^#^**
NLR-3	2.75 (0.28–17.79)	
NLR-7	1.09 (0.42–11.89)	
MLR-1	0.17 (0.00–11.08)	**<0.001** ^&,¥^
MLR-3	0.38 (0.02–2.43)	
MLR-7	0.34 (0.01–2.98)	
PLR-1	55.21 (14.36–913.51)	**<0.001** ^&,^**^#^**
PLR-3	81.94 (23.54–251.06)	
PLR-7	72.84 (24.03–117.43)	
PIV-1	129.71 (2.33–81,537.48)	**<0.001** ^¥^
PIV-3	327.86 (16.51–6086.16)	
PIV-7	362.19 (4.90–13,482)	
SII-1	178.83 (21.05–19,887.19)	**0.002** ^&,¥^
SII-3	402.70 (61.42–2650.68)	
SII-7	277.72 (76.83–2922.43)	
SIRI-1	0.588 (0.010–241.235)	**<0.001** ^&,¥^
SIRI-3	1.120 (0.109–55.836)	
SIRI-7	1.557 (0.030–71.333)	
IG-1(10^3^/µL)	0.15 (0.00–5.36)	0.063
IG-3 (10^3^/µL)	0.16 (0.01–13.80)	
IG-7 (10^3^/µL)	0.31 (0.01–9.15)	
IG%-1	0.90 (0.00–16.00)	0.105
IG%-3	1.20 (0.02–39.90)	
IG%-7	2.10 (0.10–26.70)	

^1^ Data are presented as mean ± standard deviation (SD) or median (minimum–maximum), as appropriate. Suffixes “-1,” “-3,” and “-7” denote measurements obtained on postnatal days 1, 3, and 7, respectively. Repeated-measures ANOVA with Bonferroni-adjusted post hoc comparisons were used to evaluate temporal changes across postnatal days 1, 3, and 7. ^&^ indicates comparison between day 1 and day 3; ^#^ indicates comparison between day 3 and day 7; ^¥^ indicates comparison between day 1 and day 7. * Bold *p*-values indicate statistical significance (*p* < 0.05). hsPDA: hemodynamically significant patent ductus arteriosus; NLR: neutrophil-to-lymphocyte ratio; MLR: monocyte-to-lymphocyte ratio; PLR: platelet-to-lymphocyte ratio; PIV: pan-immune inflammation value; SII: systemic immune-inflammation index; SIRI: systemic inflammation response index; IG: absolute immature granulocytes count; IG%, immature granulocyte percentage.

**Table 3:** Temporal Changes in Hematologic Parameters and Inflammatory Indices Between Postnatal Days 3 and 7 According to Treatment Response.

	Treatment Non-Responder Group, *n* = 25 ^1^	Treatment Responder Group, *n* = 46 ^1^	*p* Value
Leukocyte-3 (10^3^/µL)	10.4 (2.27–38.59)	8.76 (2.35–68.93)	0.895
Leukocyte-7 (10^3^/µL)	12.13 (6.42–47.40)	9.79 (4.62–48.02)	0.295
*p* value *	**0.005**	0.077	
Neutrophil-3 (10^3^/µL)	3.7 (0.88–31.31)	5.06 (0.67–32.67)	0.444
Neutrophil-7 (10^3^/µL)	5.55 (1.66–31.51)	3.89 (1.49–35.38)	0.389
*p* value	0.056	0.861	
Monocyte-3 (10^3^µ/L)	0.86 (0.030–7.60)	0.88 (0.18–3.52)	0.709
Monocyte-7(10^3^µ/L)	1.66 (0.050–6.21)	1.42 (0.040–6.00)	0.235
*p* value *	**0.006**	**0.010**	
Lymphocyte-3 (10^3^µ/L)	3.09 ± 1.40	2.86 ± 1.26	0.476
Lymphocyte-7 (10^3^µ/L)	3.90 ± 1.32	4.28 ± 1.32	0.256
*p* value *	**0.015**	**<0.001**	
Platelet-3 (10^3^/µL)	231.9 ± 100.6	230.9 ± 82.2	0.963
Platelet-7 (10^3^/µL)	251.6 ± 88.6	238.9 ± 83.3	0.552
*p* value	0.294	0.587	
NLR-3	2.09 (0.42–17.79)	2.15 (0.28–16.33)	0.588
NLR-7	1.2 (0.43–9.94)	1.05 (0.42–11.89	0.430
*p* value *	0.061	**<0.001**	
MLR-3	0.39 (0.02–2.43)	0.38 (0.08–1.77)	0.838
MLR-7	0.47 (0.01–2.98)	0.32 (0.01–2.47)	0.117
*p* value *	0.122	0.874	
PLR-3	75.61 (23.54–251.06)	85.56 (40.67–233.04)	0.617
PLR-7	77.25 (31.05–117.43)	72.6 (24.03–109.36)	0.691
*p* value *	0.158	**<0.001**	
PIV-3	303.38 (16.51–4702. 02)	360.41 (24.57–6086.16)	0.342
PIV-7	417.06 (12.67–9994. 71)	357.44 (4.9–13,482)	0.176
*p* value *	**<0.001**	0.658	
SII-3	343.78 (64.86–2650.68)	449.23 (61.42–2228.95)	0.248
SII-7	304.37 (93.07–2922.43)	239.62 (76.83–2247)	0.217
*p* value	0.696	0.096	
SIRI-3	1.089 (0.109–43.138)	1.285 (0.112–55.836)	0.298
SIRI-7	2.656 (0.039–55.836)	1.354 (0.03–71.333)	0.206
*p* value *	**0.010**	0.706	
IG-3 (10^3^/µL)	0.15 (0.01–13.25)	0.16 (0.01–13.8)	0.763
IG-7 (10^3^/µL)	0.31 (0.01–8.28)	0.23 (0.01–9.15)	0.291
*p* value *	**0.032**	0.439	
IG%-3	1.1 (0.016–39.9)	1.3 (0.016–39.9)	0.555
IG%-7	3.4 (0.2–26.7)	1.8 (0.1–23.7)	0.221
*p* value *	**0.044**	0.302	

^1^ Data are presented as mean ± SD or median (minimum–maximum), as appropriate. Suffixes “-1,” “-3,” and “-7” denote measurements obtained on postnatal days 1, 3, and 7, respectively. Between-group comparisons were performed using the independent samples *t*-test or Mann–Whitney U test. Within-group comparisons between day 3 and day 7 were performed using the paired-sample *t*-test or Wilcoxon signed-rank test. * Bold *p*-values indicate statistical significance (p < 0.05). NLR: neutrophil-to-lymphocyte ratio; MLR: monocyte-to-lymphocyte ratio; PLR: platelet-to-lymphocyte ratio; PIV: pan-immune inflammation value; SII: systemic immune-inflammation index; SIRI: systemic inflammation response index; IG: absolute immature granulocytes count; IG%, immature granulocyte percentage.

**Table 4:** Comparison of Δ (Day 3–Day 7) Hematologic Parameters and Inflammatory Indices According to Treatment Response.

	Treatment Non-Responder Group, *n* = 25 ^1^	Treatment Responder Group, *n* = 46 ^1^	*p* Value *
ΔLeukocyte 3–7	−3300 (−20,400–9160)	−2695 (−43,890–38,900)	0.393
ΔNeutrophil 3–7	−550 (−11,600–27,830)	0 (−33,120–23,970)	0.236
ΔMonocyte 3–7	−700 (−6180–4180)	−655 (−4600–2720)	0.448
ΔLymphocyte 3–7	−1020 (−4120–2700)	−1480 (−4950–3010)	0.147
ΔPlatelet 3–7	−32,000 (−155,000–225,000)	9500 (−257,000–282,000)	0.406
ΔNLR 3–7	0.36 (−9.41–17.2)	1.62 (−5.55–12.14)	0.250
ΔMLR 3–7	−0.08 (−2.86–1.87)	−0.04 (−1.03–1.38)	0.245
ΔPLR 3–7	12.56 (−58.87–165.65)	17.43 (−68.7–150.5)	0.427
ΔPIV 3–7	−280 (−5293–380)	−103 (−10,315–5591)	**0.044**
ΔSII 3–7	3.85 (−2375–2492)	60.49 (−1731–2069)	0.144
ΔSIRI 3–7	−0.8 (−18.27–1.08)	−0.16 (−40.29–52.9)	0.076
ΔIG 3–7	−0.12 (−8.13–6.23)	−0.045 (−7.02–13.57)	0.159
ΔIG% 3–7	−1.3 (−26.2–25.1)	−0.4 (−23.6–37.8)	0.243

^1^ Data are presented as mean ± SD or median (minimum–maximum), as appropriate. Δ values represent day 3 minus day 7 measurements. Between-group comparisons were performed using the independent samples *t*-test or Mann–Whitney U test. * Bold *p*-values indicate statistical significance (*p* < 0.05). NLR: neutrophil-to-lymphocyte ratio; MLR: monocyte-to-lymphocyte ratio; PLR: platelet-to-lymphocyte ratio; PIV: pan-immune inflammation value; SII: systemic immune-inflammation index; SIRI: systemic inflammation response index; IG: absolute immature granulocytes count, IG%, immature granulocyte percentage.

**Table 5:** Discriminative Performance of ΔPIV for Identifying Treatment Non-response in Preterm Infants with hsPDA.

	Cut-Off	AUC (95% CI)	*p*-Value	Sensitivity (%)	Specificity (%)
ΔPIV	≤110.4	0.646 (0.518–0.774)	0.026	92	37

ΔPIV was calculated as postnatal day 3 minus postnatal day 7 measurement. The cut-off value of ≤110.4 was used to identify treatment non-response. PIV: pan-immune-inflammation value; AUC: area under the receiver operating characteristic curve; CI: confidence interval.

**Table 6:** Correlations of Hematologic Parameters and Inflammatory Indices with hsPDA Status, Birth Weight, and Gestational Age on Postnatal Day 3.

Variables		hsPDA	Birth Weight (g)	Gestational Age (Weeks)
Birth weight (g)	r	0.157		
	*p*	0.192		
Gestational age (weeks)	r	0.143	0.917	
	*p*	0.234	0.000	
Leukocyte-3	r	−0.152	**0.400**	**0.401**
	*p* *	0.206	**0.001**	**0.001**
Neutrophil-3	r	−0.189	**0.368**	**0.384**
	*p* *	0.114	**0.002**	**0.001**
Lymphocyte-3	r	0.015	**0.377**	**0.359**
	*p* *	0.901	**0.001**	**0.002**
Monocyte-3	r	**−0.258**	0.172	0.166
	*p* *	**0.030**	0.152	0.166
Platelet-3	r	0.155	**0.479**	**0.305**
	*p* *	0.196	**<0.001**	**0.010**
NLR-3	r	−0.212	0.093	0.116
	*p* *	0.076	0.442	0.333
MLR-3	r	**−0.259**	0.117	0.139
	*p* *	**0.029**	0.330	0.248
PLR-3	r	0.098	0.012	−0.065
	*p*	0.417	0.921	0.589
PIV-3	r	−0.108	**0.344**	**0.318**
	*p* *	0.370	**0.003**	**0.007**
SII-3	r	−0.083	**0.325**	**0.309**
	*p* *	0.494	**0.006**	**0.009**
SIRI-3	r	−0.163	**0.274**	**0.278**
	*p* *	0.175	**0.021**	**0.019**
IG-3	r	−0.208	**0.259**	0.194
	*p* *	0.081	**0.029**	0.105
IG%-3	r	−0.135	0.061	−0.030
	*p*	0.263	0.616	0.807

hsPDA: hemodynamically significant patent ductus arteriosus; NLR: neutrophil-to-lymphocyte ratio; MLR: monocyte-to-lymphocyte ratio; PLR: platelet-to-lymphocyte ratio; PIV: pan-immune inflammation value; SII: systemic immune-inflammation index; SIRI: systemic inflammation response index; IG: immature granulocytes count, IG%, immature granulocyte percentage. Suffix “-3” denotes measurements obtained on postnatal day 3. Correlation analyses were performed using Spearman’s rank correlation coefficient. * Bold *p*-values indicate statistical significance (*p* < 0.05).

**Table 7:** Adjusted Logistic Regression Analysis of Δ CBC-Derived Inflammatory Indices (Day 3–7) Predicting Treatment Non-Response.

	*p*	OR	95% CI for OR	
			Lower	Upper
Δ Leukocyte 3–7	0.313	1.000	1.000	1.000
Δ Neutrophil 3–7	0.740	1.000	1.000	1.000
Δ Monocyte 3–7	0.241	1.000	0.999	1.000
Δ Lymphocyte 3–7	0.171	1.000	1.000	1.001
Δ Platelet 3–7	0.686	1.000	1.000	1.000
Δ NLR 3–7	0.461	0.944	0.809	1.101
Δ MLR 3–7	0.266	0.593	0.236	1.488
Δ PLR 3–7	0.575	0.997	0.985	1.009
Δ PIV 3–7	0.206	0.968	0.920	1.018
Δ SII 3–7	0.282	1.000	0.999	1.000
Δ SIRI 3–7	0.208	1.000	1.000	1.000
Δ IG 3–7	0.252	0.887	0.723	1.089
Δ IG% 3–7	0.149	0.954	0.896	1.017

Each Δ parameter (day 3 minus day 7) was entered as the predictor of interest in a separate logistic regression model, adjusted for birth weight, gestational age, and treatment type. OR: Odds ratios, represent the adjusted change in odds of treatment non-response per one-unit increase in each Δ parameter. CI: confidence interval.

## Data Availability

The data presented in this study are available on request from the corresponding author. The data are not publicly available due to privacy and ethical reasons.
